# Choledochoscope laser lithotripsy used as a remedial treatment for pancreatic duct stone basket incarceration

**DOI:** 10.1055/a-2387-4112

**Published:** 2024-09-04

**Authors:** Ping-Ping Zhang, Yan-Wei Lv, Ting Yang, Liang-Hao Hu

**Affiliations:** 1 Gastroenterology, Changhai Hospital, The Second Military Medical University, Shanghai, China

A 60-year-old woman with chronic pancreatitis and pancreatic duct stones ( Fig. 1 ) was admitted to hospital because of abdominal pain. The patient underwent three sessions of extracorporeal shock wave lithotripsy (ESWL) and was scheduled for further endoscopic stone removal ( Fig. 2 ).

**Fig. 1:**
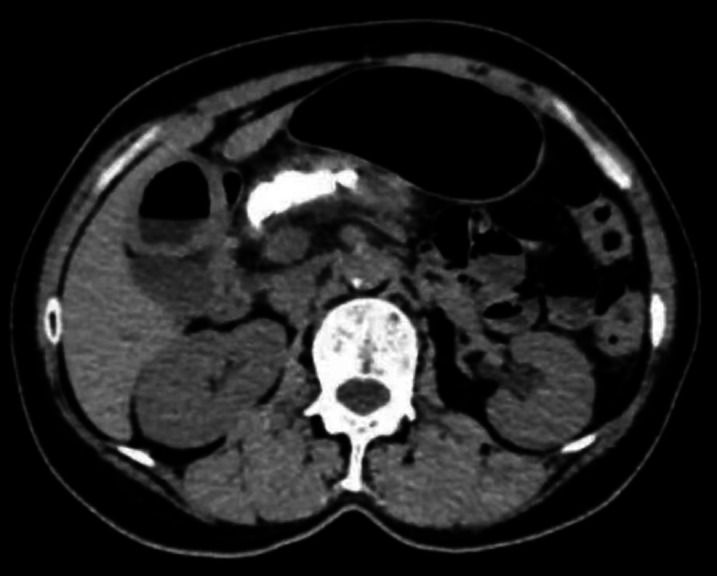
Computed tomography image showing chronic pancreatitis and pancreatic duct stones.

**Fig. 2:**
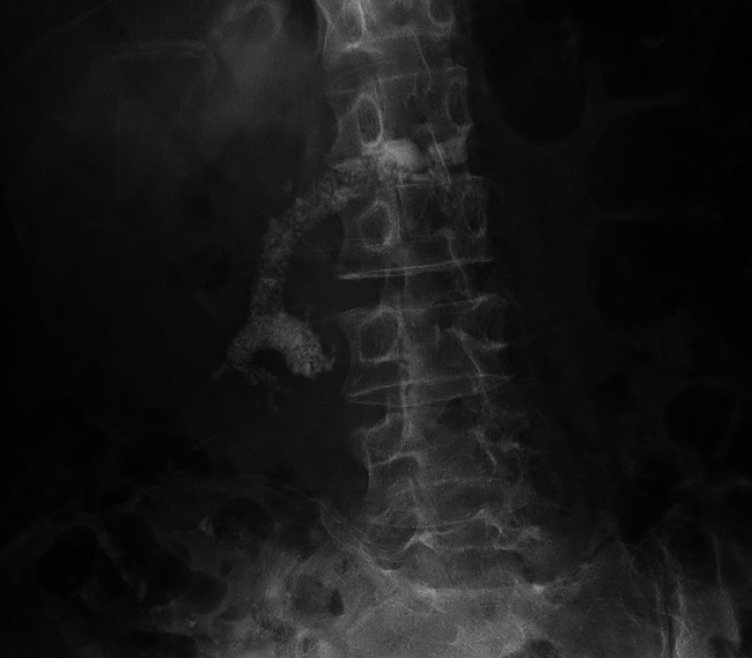
Radiographic image showing the appearance after three sessions of extracorporeal shock wave lithotripsy.

The endoscopic retrograde cholangiopancreatography (ERCP) images showed a narrowing in the head of pancreatic duct and multiple filling defects, with the shadow of a chunky stone visible in the tail of pancreas. A papillotomy was first performed, followed by balloon dilation of the narrowed pancreatic duct up to 7 mm ( Fig. 3 **a** ). A spiral stone retrieval basket (COOK) was then used to attempt to retrieve the stone; however, the basket became entrapped with the pancreatic duct stone, which was measured as having a maximum diameter of 14 mm. A dilation balloon (Boston Hurricane, 6 mm) was used to dilate the pancreatic duct, but an attempt to drag the basket out was still unsuccessful ( Fig. 3 **b** ). Subsequently, the handle of basket was cut off. A choledochoscope (SpyGlass) was then introduced via the biopsy channel of the endoscope, and a laser was introduced through the accessory channel. The U100 plus electrohydraulic lithotripsy fiber was inserted through the working tube and placed onto the surface of the stone, with intracavitary laser lithotripsy performed with a frequency of 120 Hz ( Fig. 4 ). After the stone had been fragmented into smaller pieces, the basket was pulled out under radiographic guidance. The remaining stone fragments were then successfully removed by the basket ( Video 1 ).

**Fig. 3:**
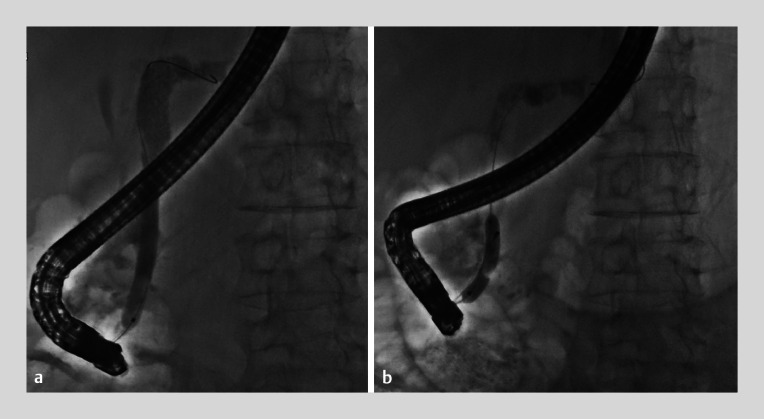
Fluoroscopic image during endoscopic retrograde cholangiopancreatography showing: **a** balloon dilation of the narrowed pancreatic duct up to 7 mm; **b** a 6-mm dilation balloon being used to dilate the pancreatic duct.

**Fig. 4:**
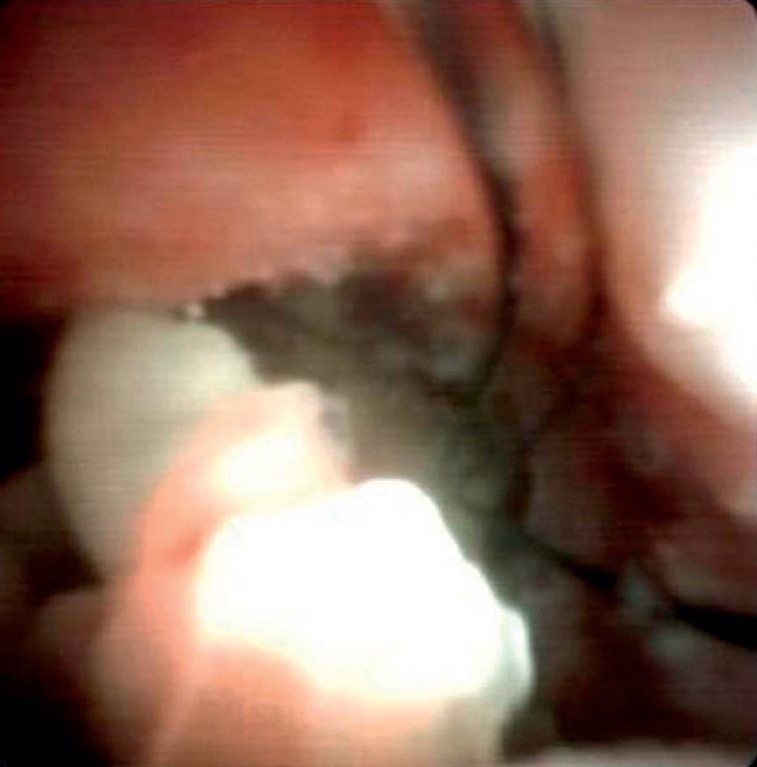
Image from a choledochoscope that had been introduced via the biopsy channel of the endoscope, with a laser introduced through the accessory channel to fragment the stone.

A basket became entrapped while ensnaring a pancreatic duct stone and attempts to drag the basket out after balloon expansion were unsuccessful; a choledochoscope laser is used to fragment the stone into smaller pieces and the basket is then pulled out and the stone fragments removed.Video 1

Stone extraction via ERCP is an effective method to treat pancreatic duct stones 1 2 . Basket incarceration is however a tricky situation that usually requires surgical treatment. In this report, we used a choledochoscope laser to fragment the stones within the incarcerated basket in the pancreatic duct, ultimately resolving the basket incarceration.

Endoscopy_UCTN_Code_TTT_1AR_2AH

## Notes

Endoscopy E-Videos https://eref.thieme.de/e-videos
*E-Videos* is an open access online section of the journal *Endoscopy* , reporting on interesting cases and new techniques in gastroenterological endoscopy. All papers include a high-quality video and are published with a Creative Commons CC-BY license. Endoscopy E-Videos qualify for HINARI discounts and waivers and eligibility is automatically checked during the submission process. We grant 100% waivers to articles whose corresponding authors are based in Group A countries and 50% waivers to those who are based in Group B countries as classified by Research4Life (see: https://www.research4life.org/access/eligibility/ ). This section has its own submission website at https://mc.manuscriptcentral.com/e-videos .
